# Compressive Large Diaphragmatic Mesothelial Cyst Mimicking a Pericardial Cyst in a Patient With Exertional Dyspnea

**DOI:** 10.1016/j.jaccas.2026.108606

**Published:** 2026-05-30

**Authors:** Alborz Sherafati, Jeremy D. Collins, Gal Tsaban, Luis F. Tapias, Jae K. Oh, Mandeep Singh

**Affiliations:** a Department of Cardiovascular Medicine, Mayo Clinic, Rochester, Minnesota, USA; b Department of Radiology, Mayo Clinic, Rochester, Minnesota, USA; c Division of Thoracic Surgery, Department of Surgery, Mayo Clinic, Rochester, Minnesota, USA

**Keywords:** computed tomography, echocardiography, shortness of breath

## Abstract

**Background:**

Diaphragmatic mesothelial cysts are rare and can be misdiagnosed as pericardial cysts. These cysts are usually asymptomatic but can cause symptoms because of compression on adjacent structures.

**Case Summary:**

A 63-year-old man presented with exertional shortness of breath. Echocardiography showed a pericardial cyst adjacent to the right heart, and stress single photon emission computed tomography was negative for ischemia. Computed tomography (CT) and magnetic resonance imaging (MRI) demonstrated a large pericardial cyst adjacent to the right coronary artery with compression on the right atrioventricular groove. He underwent surgery, which revealed a mesothelial cyst embedded in the diaphragmatic muscle.

**Discussion:**

Mesothelial cysts rarely can be misdiagnosed as pericardial cyst and can cause chest pain and shortness of breath from compressive effects. Echocardiography is usually the initial step for assessment, followed by a CT scan or an MRI, which provide more anatomical details. Surgery can be considered in symptomatic patients.

**Take-Home Messages:**

Mesothelial cysts are usually diagnosed incidentally on echocardiography, CT, or MRI. Compressive effects occur rarely with diaphragmatic mesothelial cysts and can mimic compressive pericardial cysts.

## Visual Summary

**Figure:**
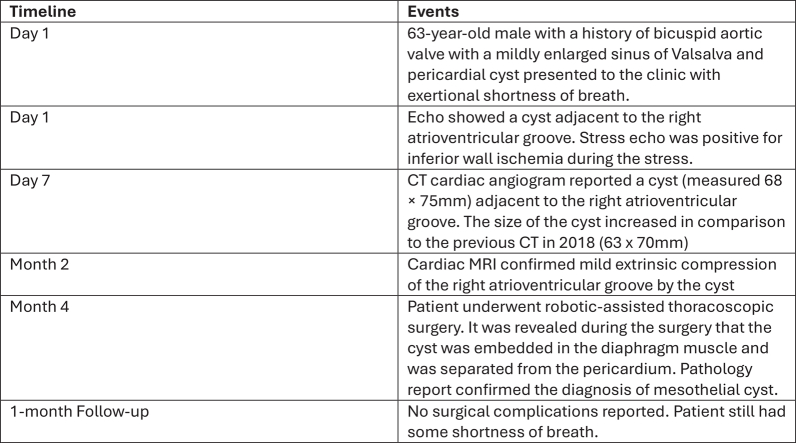

## History of Presentation

**Figure 1:**
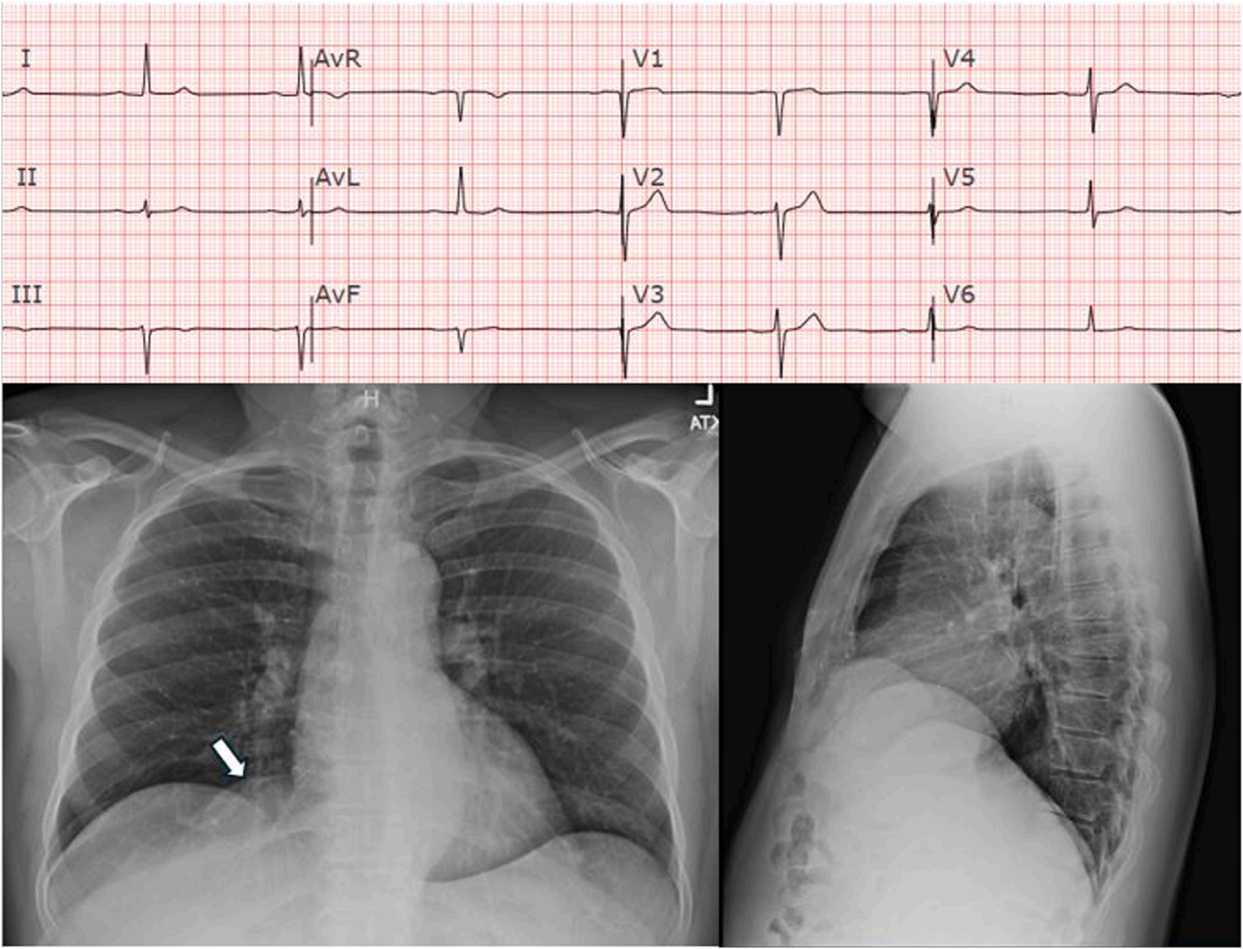
Initial Electrocardiogram and Chest X-Ray (Top) Electrocardiogram showed sinus bradycardia with no abnormalities. (Bottom) Posteroanterior and lateral chest x-ray showed an abnormal soft tissue density contour at the right cardiophrenic (arrow). The lateral view demonstrates an additional distinct contour in addition to the anterior peaked right hemidiaphragm, the “third mound.”

A 63-year-old man presented with exertional shortness of breath for 2 years which had worsened in the past 6 months. He denied chest pain, dizziness, or syncope. His blood pressure was 117/50 mm Hg, and his heart rate was 55 beats/min. The physical examination and initial laboratory results were unremarkable. The initial electrocardiogram showed sinus bradycardia with no ischemic changes or other abnormalities (Figure 1). Chest x-ray showed an abnormal contour to the right inferior cardiomediastinal border in the paracardiac region (Figure 1).

## Past Medical History

The patient had history of hypertension, dyslipidemia, type 2 diabetes, obstructive sleep apnea, and bicuspid aortic valve with a mildly enlarged sinus of Valsalva. He was also diagnosed with a 67 × 66 mm pericardial cyst in 2016. No history of previous surgeries or interventions was noted.

## Differential Diagnosis

Considerations included heart failure, coronary artery disease, and aortic regurgitation or stenosis in the setting of the bicuspid aortic valve.

## Investigations

**Figure 2:**
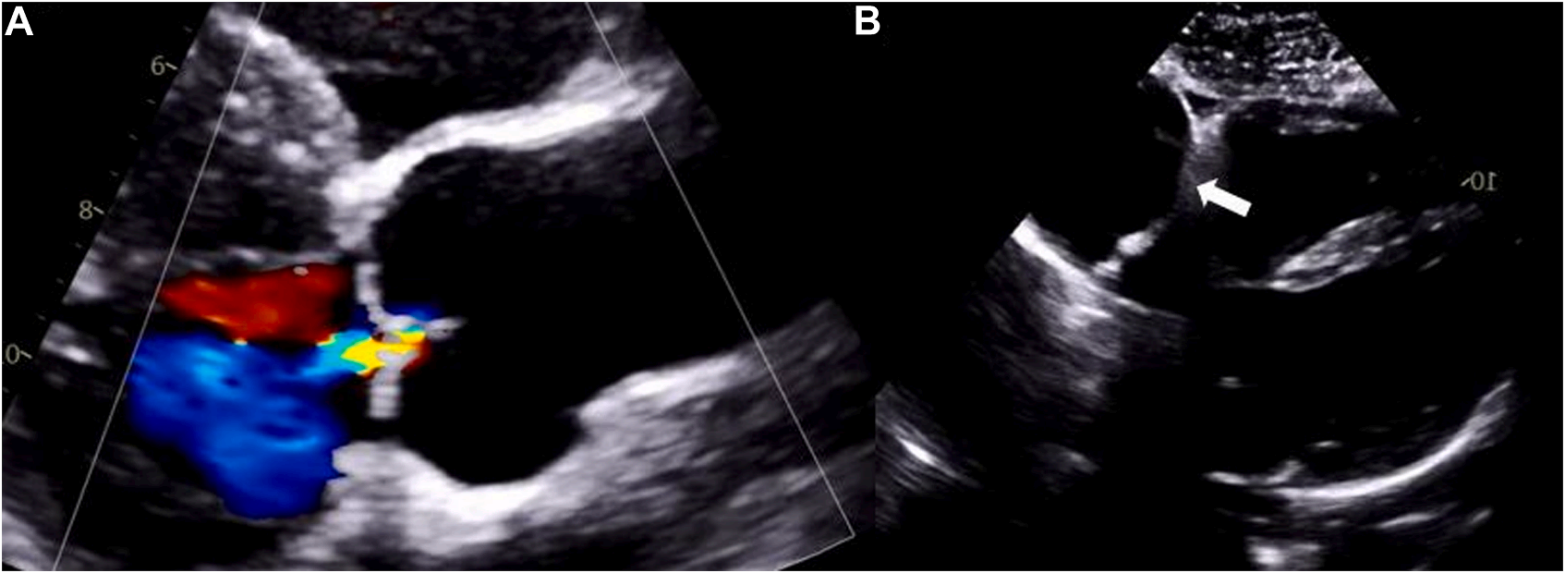
Transthoracic Echocardiography Echocardiogram revealed an enlarged sinus of Valsalva with mild aortic regurgitation (A) and no aortic stenosis. (B) A cyst was detected adjacent to the right atrioventricular groove (arrow).

**Figure 3:**
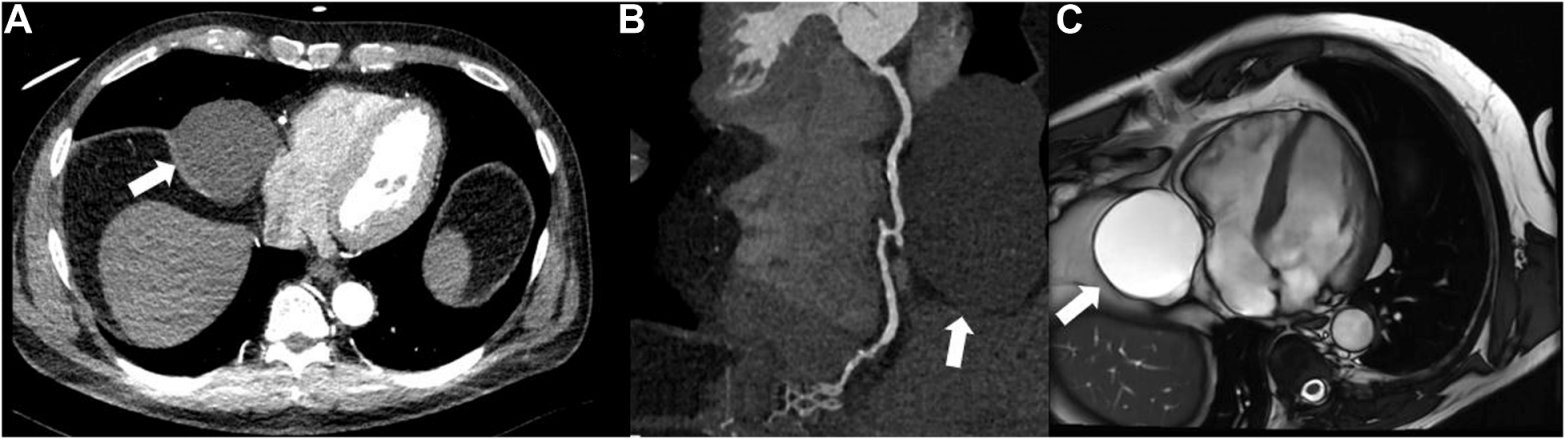
Coronary Computed Tomography and Magnetic Resonance Imaging Coronary computed tomography showed the presence of a large cyst (arrow) adjacent to the (A) right atrioventricular groove and the (B) right coronary artery. There was mild atherosclerosis of the right coronary artery. (C) Cardiac magnetic resonance imaging confirmed mild extrinsic compression of the right atrioventricular groove by the cyst (arrow).

Transthoracic echocardiography showed a normal ejection fraction, with no regional wall motion abnormalities, Sievers type I bicuspid aortic valve, sinus of Valsalva enlargement (4.5 cm), and normal ascending aortic dimensions with mild aortic regurgitation (Figure 2). A cyst was visible adjacent to the right heart. Stress echocardiography revealed new hypokinesia of the inferior and inferoseptal walls during the stress (Videos 1 and 2). However, his stress single photon emission computed tomography (SPECT) was negative for ischemia. Computed tomography (CT) cardiac angiography reported a cyst adjacent to the right atrioventricular groove. The size of the cyst increased in comparison to the CT cardiac angiography performed in 2018 (63 × 70 mm) and now measured 68 × 75 mm (Figure 3). CT angiography also showed mild stenosis of the right coronary artery. Cardiac magnetic resonance imaging (MRI) confirmed mild extrinsic compression of the right atrioventricular groove by the cyst (Figure 3).

## Management

**Figure 4:**
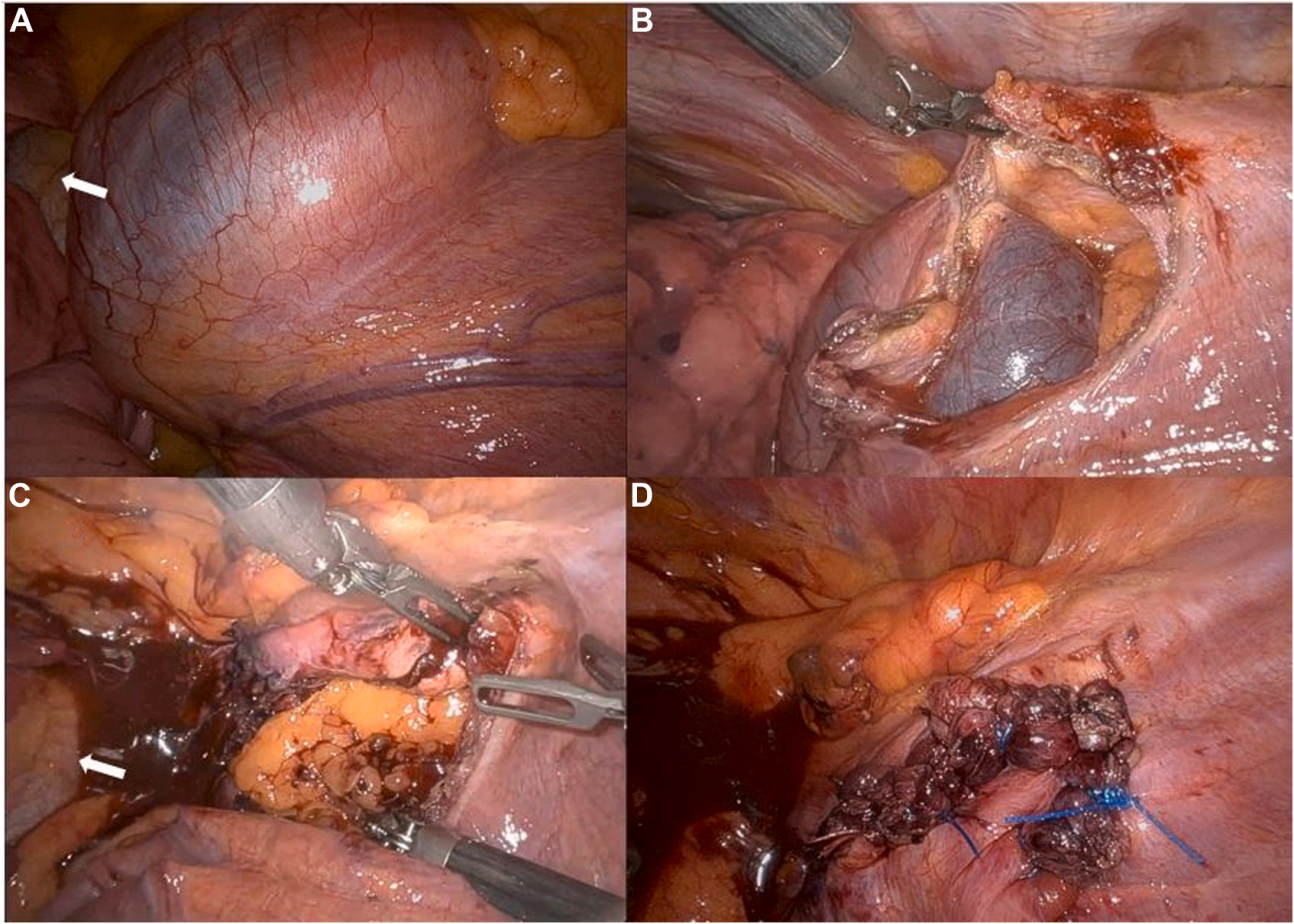
Surgical Removal of the Diaphragmatic Mesothelial Cyst (A) Initial view of the right diaphragm, showing the cyst embedded in the diaphragm muscle fibers. The right lung is seen to the left of the image. The pericardium is seen at a distance toward the left of the image (arrow). (B) View of the cyst after partially opening the diaphragm. The right lung is to the left of the image. (C) Resultant defect in the diaphragm after excision of the cyst. The pericardium is seen to the left (arrow). (D) Final view of the repair of the diaphragm.

**Figure 5:**
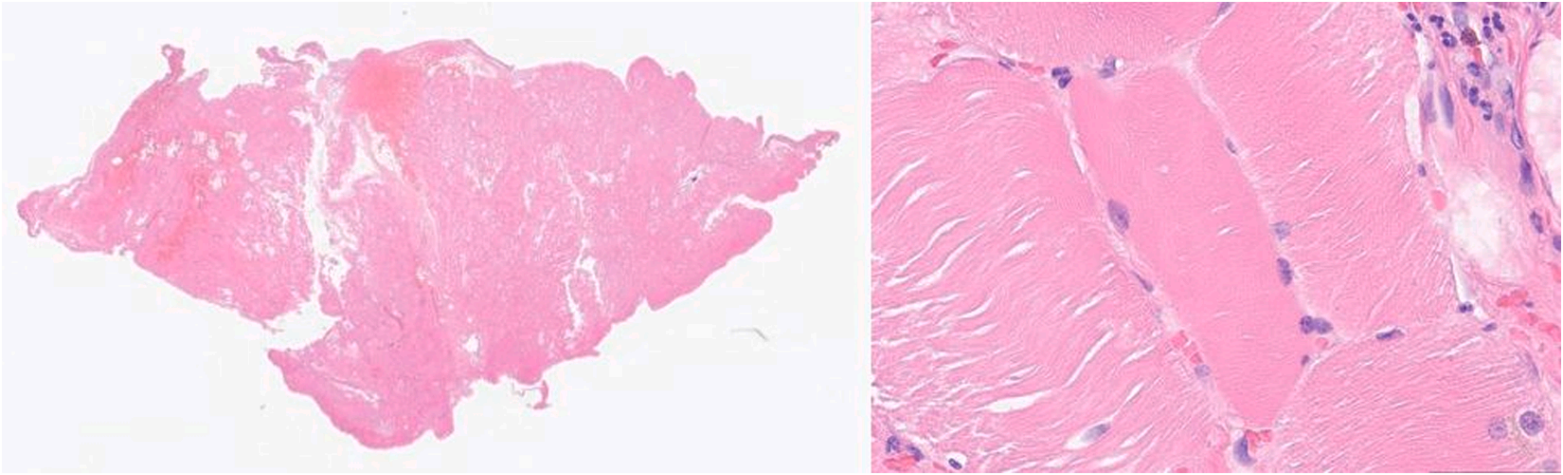
Histopathologic Examination Confirming the Presence of the Mesothelial Cyst

The patient had exertional shortness of breath, and there was inferior and inferoseptal hypokinesia on stress echocardiography, compatible with right coronary artery territory ischemia. The size of the cyst increased as compared to the previous assessment, was adjacent to the right coronary artery, and had a compressive effect on the right atrioventricular groove. Based on the location and anatomical characteristics, a diagnosis of pericardial cyst was made. With this background, and using a heart team approach, the patient underwent robotic-assisted thoracoscopic surgery. It was revealed during the surgery that the cyst was embedded in the diaphragm muscle and was separated from the pericardium (Figure 4). The cyst was completely removed and the diaphragm was repaired. Pathology report confirmed the diagnosis of mesothelial cyst (Figure 5).

## Outcome and Follow-Up

The postoperative course was uneventful, and the patient was discharged with no complications. The patient still had some shortness of breath at the 1-month follow-up.

## Discussion

Diaphragmatic cysts are rare and are classified as bronchogenic or mesothelial depending on the cyst lining cells.^1^ Diaphragmatic mesothelial cysts are usually found incidentally during imaging.^1^ Because they are rare and it is difficult to locate them anatomically, they can be misdiagnosed as pericardial cysts.^2^ Pericardial cysts are rare, with an incidence rate of about 1 in 100,000.^3^^,^^4^ Similar to mesothelial cysts, pericardial cysts are asymptomatic in up to three-fourths of patients and are diagnosed incidentally on a chest x-ray or CT scan.^3^ Larger cysts can have compressive effects on adjacent structures, resulting in symptoms such as chest pain, shortness of breath, or palpitations.^3^^,^^4^ Both types of cysts are mostly congenital, consisting of a layer of mesothelial cells, connective tissue, and elastic fibers.^1^^,^^3^^,^^4^

Diaphragmatic mesothelial cysts are common on the right side, between the diaphragm and the right posterior segment of the liver^1^ (Table 1). Thus, the most common misinterpretation is mistaking them with a hepatic cyst.^1^ The typical location of pericardial cysts is also at the right cardiophrenic angle.^5^ The unusual location of the mesothelial cyst above the right side of the diaphragm in our patient mimicked a pericardial cyst.

**Table 1:** Comparison Between Diaphragmatic Mesothelial Cysts and Pericardial Cysts

	Diaphragmatic Mesothelial Cyst	Pericardial Cyst
Location	Mostly below diaphragm and above the right posterior segment of liver	Mostly costophrenic angle (more commonly on the right side)
Etiology	Congenital	Mostly congenital but can be acquired secondary to rheumatoid pericarditis, trauma, cardiac surgery, or even viral pericarditis
Imaging findings	Nonenhancing, homogenous lesion, bilobulate shape An obtuse angle along the diaphragm with the lung parenchyma suggests extrapericardial location	Nonenhancing, homogenous lesion, unilocular and oval-shaped Local mass effect on both the lung and heart suggests pericardial involvement
Treatment	Usually conservative; other options include surgical excision, percutaneous drainage, and sclerotherapy	Usually conservative; other options include surgical excision, percutaneous drainage, and ethanol sclerosis

Initial suspicion of the presence of a cyst could be on a simple chest x-ray, where an enlarged right heart border contour is detected.^5^ We herein describe the 3- mound sign that highlights the third dome formed by the cyst (an additional distinct contour to the anterior right hemidiaphragm on the lateral view of the chest x-ray).^5^^,^^6^ Transthoracic echocardiography is the initial imaging modality performed in symptomatic, as well as in patients with incidental findings, although its sensitivity is not high.^6^ Echocardiography is helpful in differentiating the cyst from pericardial fat pad, localized pericardial effusion, and solid tumors.^5^ A CT scan or an MRI is usually performed after detecting the cyst on the echocardiogram.^6^ CT and MRI provide detailed anatomical visualization, including compressive effects,^5^ with MRI being superior to CT in identifying the fluid nature of the cyst.^6^

Treatment strategy depends on the presence of symptoms and increase in the size of the cyst over time. Asymptomatic individuals with no/minimal interval increase in the size of the cyst are managed conservatively with follow-up imaging every 1 to 2 years, whereas surgery is reserved for symptomatic patients, enlarging cysts, and the presence of complications, including infection.^3^^,^^4^^,^^6^ Endoscopic resection is preferred to open resection given faster recovery and less postoperative pain.^4^

In our patient, the cyst was initially suspected as pericardial on the chest x-ray and echocardiography, and its location adjacent to the right atrioventricular groove was confirmed by the CT scan. The compressive effect of the cyst was best visualized on MRI. Our patient had new-onset shortness of breath for 2 years, and his symptoms were getting worse, resulting in a limitation in his physical activity. His cyst was adjacent to the right coronary artery, with a compressive effect on the right atrioventricular groove on MRI. As the cyst had increased in size and the location of the compression was compatible with the regional wall ischemia on stress echocardiogram, based on a heart team approach, removal by surgery was considered the next best step.

**Figure 6:**
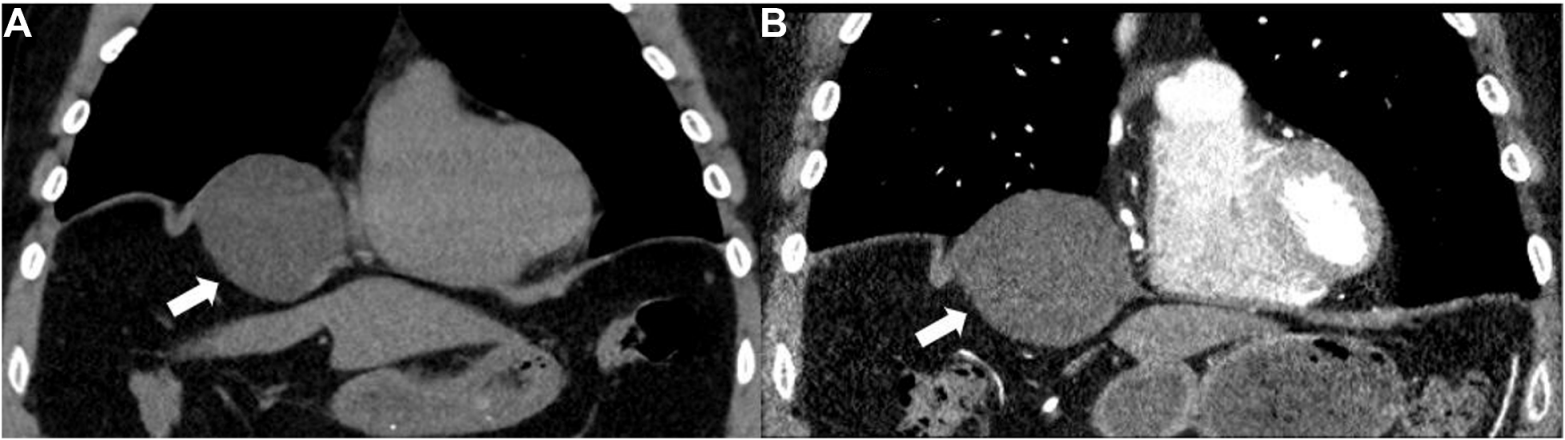
Computed Tomography Coronal View Computed tomography coronal view of the diaphragmatic mesothelial cyst (arrow) in (A) 2018 and (B) 2025. The lesion has grown slightly overtime. There is cranial displacement of the diaphgram along the lateral edge (rightward). This favors extrapericardial and diaphragmatic rather than pleural source of origin.

In retrospect, MRI signal characteristics, shape, morphology, or location alone are insufficient to distinguish between a pericardial cyst and a diaphragmatic mesothelial cyst. As the cyst enlarges, looking carefully at local mass effect is an additional consideration. In the presented case, there is an obtuse angle along the diaphragm with the lung parenchyma, which suggests extrapericardial location^7^ (Figure 6). However, the local mass effect on both the lung and heart raised the question of pericardial involvement. The lesion margin angle to the lung in this case (Figure 3A) raises the question of an extrapericardial source of origin, and the lesion location along the diaphragm with interval growth affecting the diaphragm and lung more than the heart suggests a paracardiac rather than pericardial origin (Figure 6). The margin along the lateral diaphragm favors the diaphragm over the pleura. A complementary 3-dimensional imaging approach (CT, or specific sequences on MRI), as in this case, is required to catch such details. Clear visualization of the pericardial layers separate from the cyst is challenging when the lesion is in a paracardiac location with mass effect on the cardiac structures; such a distinction on imaging would be definitive in the correct identification of an extrapericardial source of origin.

## Conclusions

We have described a patient with exertional shortness of breath, with evidence of inferior and inferoseptal myocardial ischemia on stress echocardiography and an enlarged cyst with compressive effect on the right atrioventricular groove and possibly the right coronary artery. It was initially thought to be a pericardial cyst. However, it was revealed during the surgery that it was a diaphragmatic mesothelial cyst. Mesothelial cysts are usually diagnosed incidentally. However, if such patients develop symptoms of myocardial ischemia, including chest pain and shortness of breath, it is reasonable to assess the possible compressive effect of the cyst on the coronary arteries. Surgical removal of the cyst could be considered in such patients, which could result in an improvement in myocardial ischemia and the patient's symptoms.

## Funding Support and Author Disclosures

The authors have reported that they have no relationships relevant to the contents of this paper to disclose.

**Take-Home Messages**

• Diaphragmatic mesothelial cysts can be mistaken for a pericardial cyst. Multimodality imaging can hint at the source, but it is not definitive.
• Patients with the compressive effect of the cyst on the coronary arteries, based on symptoms and the presence of objective myocardial ischemia in the relevant territory, need surgical intervention.

## Appendix

Video 1 Apical 2-Chamber View Showing Inferior Wall Hypokinesia

Video 2 Apical 2-Chamber View Showing Inferior Wall Hypokinesia at Peak Stress
